# Correction to: Long non-coding RNA LINC00968 attenuates drug resistance of breast cancer cells through inhibiting the Wnt2/β-catenin signaling pathway by regulating WNT2

**DOI:** 10.1186/s13046-021-01991-x

**Published:** 2021-06-22

**Authors:** Dian-Hui Xiu, Gui-Feng Liu, Shao-Nan Yu, Long-Yun Li, Guo-Qing Zhao, Lin Liu, Xue-Feng Li

**Affiliations:** 1 Department of Radiology, China-Japan Union Hospital of Jilin University, Changchun, 130033 People’s Republic of China; 2 Department of Anesthesiology, China-Japan Union Hospital of Jilin University, No. 126, Xiantai Street, Changchun, 130033 Jilin Province People’s Republic of China

**Correction to: J Exp Clin Cancer Res 38, 94 (2019)**

**https://doi.org/10.1186/s13046-019-1100-8**

Following publication of the original article [1], the authors identified some minor errors in image typesetting in Fig. 6; specifically the flow cytometry apoptosis experiment detailed in Fig. 6a.

The corrected figure is given below. The correction does not have any effect on the results or conclusions of the paper. The original article has been updated.

**Fig. 6:**
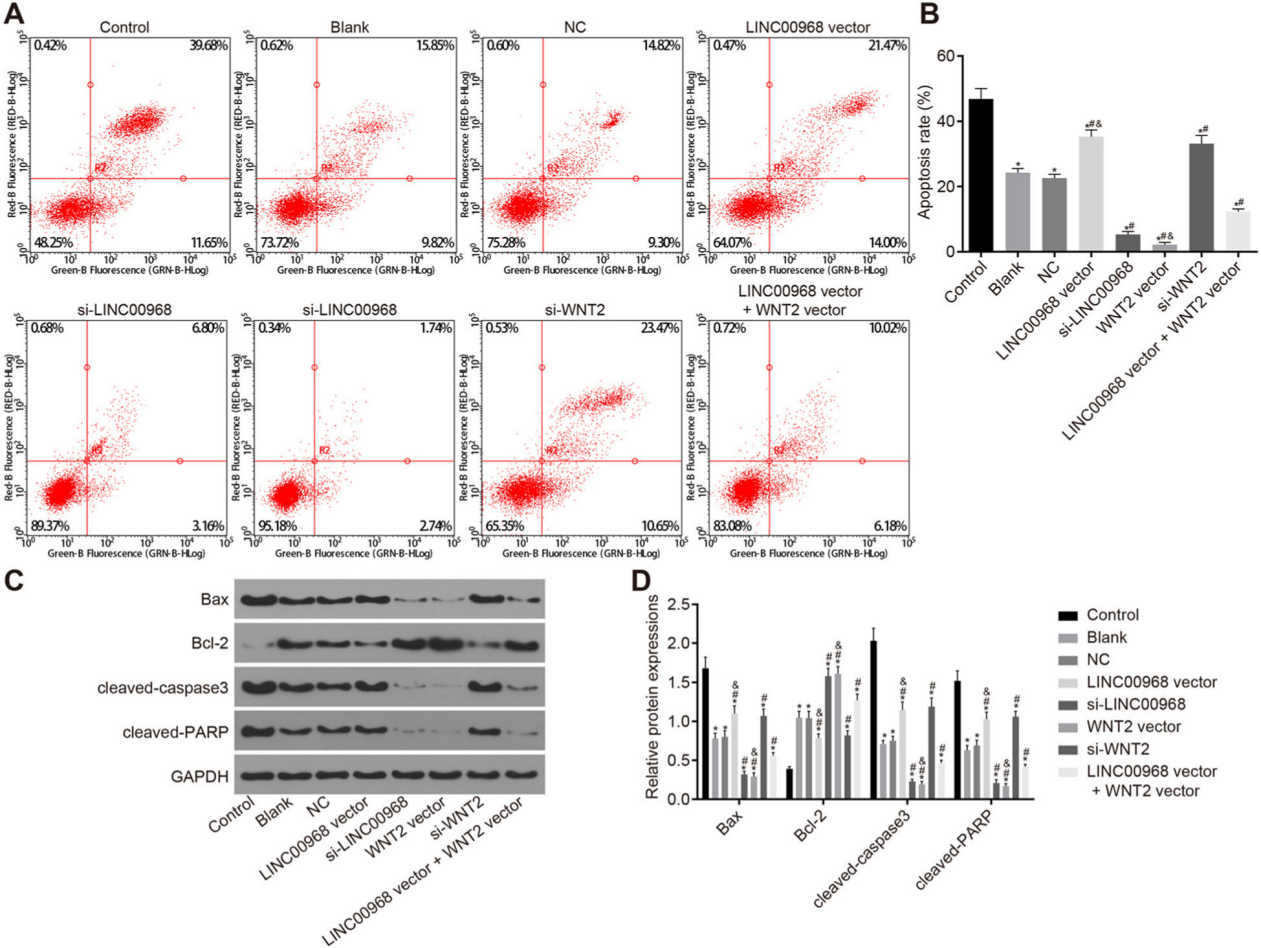
Overexpressed LINC00968 or silenced WNT2 contributes to promoted apoptosis of breast cancer cells. **a** the apoptosis of MCF-7 cells after transduction in each group as analyzed by flow cytometry. **b** the cell apoptosis rate in each group. **c** protein bands of Bax, cleaved-PARP, cleaved-caspase3 and Bcl-2 in MCF-7/ADM cells after transduction in each group as measured by Western blot analysis. **d** the protein levels of Bax, cleaved-PARP, cleaved-caspase3 and Bcl-2 in each group. *p < 0.05 vs. the control group; ^#^*p* < 0.05 vs. the blank and NC groups; ^&^*p* < 0.05 vs. the LINC00968 vector + WNT2 vector group

## References

[1] Xiu DH, Liu GF, Yu SN, Li LY, Zhao GQ, Liu L, Li XF (2019). Long non-coding RNA LINC00968 attenuates drug resistance of breast cancer cells through inhibiting the Wnt2/β-catenin signaling pathway by regulating WNT2. J Exp Clin Cancer Res.

